# Improved quality of life among adolescents with attention-deficit/hyperactivity disorder is mediated by protective factors: a cross sectional survey

**DOI:** 10.1186/s12888-015-0491-0

**Published:** 2015-05-07

**Authors:** Jorun Schei, Torunn Stene Nøvik, Per Hove Thomsen, Marit S Indredavik, Thomas Jozefiak

**Affiliations:** 1 Regional Centre for Child and Youth Mental Health and Child Welfare, Norwegian University of Science and Technology, Pb 8905 MTFS, 7491 Trondheim, Norway; 2 Department of Child and Adolescent Psychiatry, St. Olavs Hospital Trondheim University Hospital, Pb 6810 Elgeseter, 7433 Trondheim, Norway; 3 Psychiatric Hospital for Children and Adolescents, Aarhus University Hospital, Skovagervej 2, 8240 Risskov, Denmark

**Keywords:** ADHD, Adolescence, Coexisting problems, Protective factors, QoL

## Abstract

**Background:**

The aim of this study was to assess the role of protective factors as mediators and/or moderators of the relationship between coexisting emotional and conduct problems and quality of life (QoL) among adolescents with attention-deficit/hyperactivity disorder (ADHD).

**Methods:**

The sample consisted of 194 adolescents with ADHD. Participants completed measures of individual competencies, family cohesion and social support, and QoL. Coexisting emotional and conduct problems were assessed using the Strength and Difficulties Questionnaire.

**Results:**

Individual competencies and social support mediated the association between emotional and conduct problems and QoL. Family cohesion was associated with both emotional and conduct problems. No moderating effects of protective factors and coexisting problems were found.

**Conclusions:**

The assessment of individual competencies, social resources, and family cohesion may identify potential treatment goals for adolescents with ADHD and coexisting problems, and may contribute to improvements in QoL.

## Background

Attention-deficit/hyperactivity disorder (ADHD) is a heterogeneous and composite disorder [1] that is characterized by symptoms of inattention, hyperactivity, and impulsivity, which affect functioning in academic, social, and family contexts [2,3]. Adolescents with ADHD and coexisting emotional and conduct problems exhibit an increased risk of criminality [4], substance abuse [5], psychiatric admissions [6], premature death [7], poorer psychosocial functioning [8], and quality of life (QoL) [9] than do adolescents with ADHD without coexisting problems. Although ADHD is considered a strongly hereditary disorder [10], environmental factors in early life may also be important risk factors for the development of this condition [11]. The literature shows that individual and environmental factors may interact with genes to affect brain maturation among individuals with ADHD during childhood and adolescence [12]. Thus, the investigation of risk and protective factors that are important for outcome among adolescents with ADHD is critical. Although the impact of coexisting disorders on QoL has been documented among adolescents with ADHD [9,13], little is known about which protective factors, if any, mediate and/or moderate this relationship.

Protective factors include both individual and environmental factors, and can be measured [14]. These factors lessen child maladjustment after life events [15]. Individual factors include competencies such as structured style, social competence, and personal competence. Structured style relates to executive functioning skills, e.g., planning, organization, and goal orientation. Environmental factors include social resources and family cohesion. Social resources address social support, such as having friends. A substantial proportion of ADHD patients have deficits in executive functioning tasks, which could be a causal factor for ADHD symptoms in a subset of patients [16,17]. ADHD patients also have poor social functioning (i.e., possessing a positive social orientation) and personal competence (i.e., self-esteem and self-efficacy) [18]. Poor social competence has been associated with conduct and emotional problems [19], including ADHD [20], and poor self-esteem plays a role in the association between social phobia and depression [21].

Coexisting emotional and conduct problems are risk factors for an unfavorable outcome for adolescents with ADHD [4-7,22]. We recently found that coexisting emotional and conduct problems in adolescents with ADHD were associated with low self-reported family functioning [23]. Rinsky and Hinshaw [24] found that childhood planning abilities predicted comorbid emotional and behavioral problems and social functioning in adolescence. The authors [24] reported that social functioning mediated the relationship between planning abilities and comorbidities, and that comorbidity mediated the relationship between planning abilities and social functioning.

QoL is a multidimensional concept and has various definitions [25]; nevertheless, it is commonly referred to as subjectively perceived well-being and satisfaction within several life domains [26], such as physical and mental health, friends, family, school, and time alone. Among a clinical sample of children with various diagnoses, it was shown that it is possible to improve QoL without reducing symptoms, which demonstrates the importance of assessing QoL [27]. A large European study [13] that assessed multiple factors that are possibly associated with QoL among children and adolescents with ADHD found that the presence of peer problems and emotional problems was most strongly associated with poor QoL outcomes. However, to date, few studies have addressed why adolescents with ADHD and coexisting emotional and conduct problems have impaired QoL [9].

For children and adolescents with ADHD, medical treatment is one of the major options to decrease ADHD symptoms and improve psychosocial functioning and QoL [25,28-30]. However, the complex nature of ADHD means that several channels of intervention are needed, especially in comorbid cases [31]. These interventions might include peer and friendship coaching [32] and organizational training [33].

Previous research has focused on the direct relationships between psychopathology and QoL [9,13]. However, protective factors may mediate and/or moderate the relationship between coexisting emotional problems and conduct problems and be considered as targets of treatment [9,34]. Therefore, we aimed to assess the mediating and moderating effect of individual competencies, family cohesion, and social resources on the relationship between coexisting emotional problems and conduct problems and QoL. By exploring these relationships in a sample of adolescents with ADHD, we hypothesized that the direct effect between emotional and conduct problems and QoL is mediated by individual competencies, family cohesion, and social resources, which implies that better protective factors decreased the negative effect of risk factors on QoL. Our second hypothesis was that adolescents in the sample who were receiving medication have fewer emotional and conduct problems and better QoL. Finally, our third hypothesis was that protective factors moderate the effect of coexisting problems and ADHD symptom level on QoL. We included key covariates in the direct and final path model (age, sex, level of ADHD symptoms, and medication) to determine the specificity of the protective factors. Associations in the path model were also adjusted for all other variables included in it.

## Methods

### Clinical sample

This study was part of The Health Survey performed by the Department of Child and Adolescent Psychiatry (CAP) at St. Olav’s University Hospital in Norway. This was a cross-sectional study of a defined clinical population. The catchment area was a county in Norway with 303,664 inhabitants, which includes urban and rural areas. The Department of CAP at the University Hospital covers all inhabitants in the county. The inclusion criteria were as follows: referred adolescents, age between 13 and 18 years, and presence of at least one attendance to the clinic between February 15, 2009 and February 15, 2011. Exclusion criteria were as follows: major difficulties in answering the questionnaire because of psychiatric state, cognitive dysfunctions, or lack of sufficient language skills. Emergency patients were invited to take part once stabilized. Among the 1,648 eligible and invited adolescents, 717 (43.5%) participated in the CAP survey. This survey and the representativeness of the sample have been described in detail previously [19]. Of the 717 participants, 243 adolescents were diagnosed with ADHD. Patients with a missing Strengths and Difficulties Questionnaire (SDQ) [35,36] were excluded from the study (*n* = 49), leaving 194 participants in the present study (final response rate, 34.8%): 87 girls and 107 boys.

### Procedure

Newly referred patients and patients who were already enrolled in the CAP clinic received oral and written invitations to participate in the study at first attendance after commencement of the project. The participating adolescents responded to an electronic questionnaire and data were collected from clinical charts. The ADHD rating scale was collected from the period of assessment prior to the initiation of medical treatment. Parents also responded to a questionnaire with items related to educational level.

### Measures

#### Sociodemographic information

The parents of the participants completed a demographic form with information about age, sex, and socioeconomic status (SES). The highest educational level of parents on an 8-point Hollingshead scale was used to estimate SES [37].

#### Clinical diagnosis

Diagnoses were collected from clinical charts and were established according to the *International Statistical Classification of Diseases and Related Health Problems* (10th revision (*ICD-10*) [38] multiaxial diagnostic system (i.e., axes I–VI). All diagnoses were made by a clinical psychologist or a child and adolescent psychiatrist based on the available clinical information. The CAP clinic’s standardized procedure for the assessment and diagnosis of hyperkinetic disorders is based on the national guideline for the assessment and treatment of ADHD [39]. This guideline, similar to other established ADHD guidelines [40], requires a clinical diagnostic interview based on ADHD as described in the *Diagnostic and Statistical Manual of Mental Disorder* 4th edition, text revision *(DSM-IV-TR)* [41], possible coexisting disorders, and a somatic assessment; it recommends the use of questionnaires filled out by the adolescent, parent, and teacher to obtain ADHD symptom scores (ADHD rating scale). The *ICD-10* diagnosis of hyperkinetic disorder is referred to as ADHD in this study. The diagnostic criteria for hyperkinetic disorder are nearly identical to the criteria for ADHD combined type in the *DSM-IV-TR* [41], however, specifiers such as mainly attention problems or hyperactivity/impulsivity problems are not utilized in the *ICD-10*. A recent study of adults showed that *DSM-IV-TR* ADHD inattentive and hyperactive-impulsive types are less likely to qualify for a diagnosis of hyperkinetic disorder [42]. Coexisting disorders from clinical charts were not used in the present study.

#### Medication

nformation about medical treatment was collected from clinical charts, including prescribed medicines (methylphenidates, amphetamines, or atomoxetine). Data from the clinical charts verified that the patients had entered a stable phase with a documented effect of the medication.

#### ADHD Rating Scale IV (ADHD-RS)

ADHD symptoms were measured using the ADHD-RS, parent version [43]. The instrument contains 18 items that address ADHD symptoms based on the DSM-IV criteria. The items are measured on a 5-point scale, in which higher scores reflect higher frequencies of symptoms. The scale is organized into two sections, each with its own sum score. One reflects symptoms of inattention, whereas the other reflects hyperactivity and impulsivity.

#### Strengths and Difficulties Questionnaire (SDQ)

Coexisting problems were measured using the Norwegian version [35] of the SDQ [36]. This clinical and research instrument contains 25 items that address emotional and behavioral problems, as well as personal strengths [36]. The SDQ subscales have shown satisfactory to good internal consistency, and the stability of the basic psychometric properties of the SDQ has been demonstrated across clinical samples [44]. In the present study, the three subscales, Emotional Problems, Conduct Problems, and Hyperactivity/Inattention, were used as indicators of latent construct emotional problems, conduct problems, and hyperactivity/inattention problems, respectively. The SDQ adolescent self-report exhibited satisfactory construct validity and internal consistency in a study performed by the original author; the Cronbach alphas of the self-report were as follows: total difficulties, 0.80; emotional problems, 0.66; conduct problems, 0.60; and hyperactivity/inattention, 0.67 [45]. Van Roy et al. [35] found the SDQ self-report to be appropriate for children and adolescents aged 10–19 years. Another study performed by the same authors divided the sample according to the following age groups: 10–13 (preadolescent), 13–16 (early adolescent), and 16–19 (late adolescent) years. The early and late adolescent groups had the following Cronbach alphas, respectively: emotional problems, .71 and .70; conduct problems, .59 and .54; and hyperactivity, .65 and .66 [46].

#### Resilience Scale for Adolescents (READ)

Protective factors were measured using the READ, which is a 23-item self-report scale that is based on a 5-point Likert scale [14]. Higher scores on the READ reflect lower degrees of resilience. The construct and convergent validity were adequately assessed. The READ is based on the Resilience Scale for Adults [47], and consists of the same five subscales: 1) Personal Competence, 2) Social Competence, 3) Structured Style, 4) Family Cohesion, and (5) Social Resources. The items on three subscales (i.e., Personal Competence, Social Competence, and Structured Style) were used as indicators of the latent concept individual competencies; items from the Family Cohesion and Social Resources subscales were used as indicators of two latent environmental protective factors. In the current study, READ showed satisfactory psychometric characteristics for the total scale (α = 0.98) and for the three subscales: Personal Distributions (α = 0.97), Family Cohesion (α = 0.89), and Social Resources (α = 0.91).

#### Inventory of Life Quality in Children and Adolescents (ILC)

QoL was measured using the Norwegian version [48] of the ILC [49,50]. This 7-item self-report inventory includes one item for global evaluation of QoL and six items that address the child’s physical and mental health, perception of activities when alone, perceived relationships with friends and family, and functioning in school. Each item uses a 5-point Likert scale, with lower scores reflecting a higher QoL. In the present study, the seven items were used as indicators of the latent concept QoL. Reliability testing in the present study indicated good internal consistency (α = 0.94). The construct validity of the ILC is also satisfactory [50].

### Ethics

Written informed consent was obtained from adolescents and parents prior to inclusion, according to the study procedures of the CAP survey. Study approval was given by the Regional Committees for Medical and Health Research Ethics (CAP survey reference number: 4.2008.1393; present study: 2011/1772) and by the Norwegian Social Science Data Services (CAP survey reference number: 19976).

### Statistical analyses

**Figure 1:**
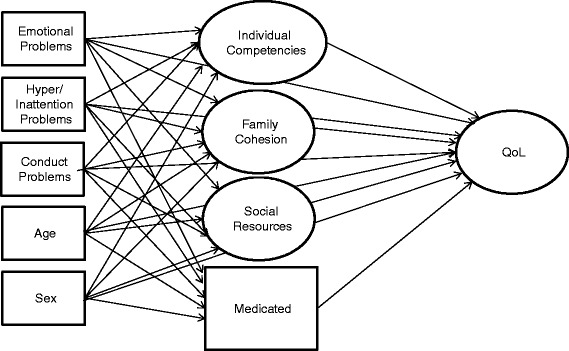
The tested mediator model.

Statistical analyses were conducted using SPSS version 19 and Mplus version 7 [51]. The frequency of missing values was between 2% and 5%. All missing values were imputed using full information maximum likelihood. We used a confirmatory factor analysis of the READ to validate the three subscales. The following indexes were used to assess the goodness of fit of the models [52]: the chi-squared test, the comparative fit index (CFI), the Tucker–Lewis index (TLI), and the root mean square error of approximation (RMSEA). Regarding CFI and TFI, values above 0.95 are considered indicators of good fit; for RMSEA, values below 0.06 are considered indicators of good fit [53]. The structural equation model was estimated using the weight least square parameter estimator (WLSMV), because of the categorical nature of the indicators. A saturated structural model was tested, in which all latent variables were regressed on each other and on the observable scales (see Figure 1). In addition to the mediator model, we tested if protective factors interacted with the Emotional Problems, Conduct Problems, and Hyperactivity/Inattention SDQ scales in the model. Two-tailed tests (*p* < 0.05) were used to measure statistical significance.

## Results

### Descriptive data of the sample

See Table 1. The mean scores of the ADHD-RS were similar to those reported for another Norwegian clinical sample of adolescents with ADHD [54].

**Table 1:** **Descriptive data of the study sample: 194 adolescents with ADHD**

	Mean (SD)	*n* (%)
Age	15.48 (1.71)	
SES	4.78 (1.82)	
SDQ Emotional Problem scale	4.23 (2.76)	
SDQ Conduct Problem scale	3.01 (1.88)	
SDQ Hyperactivity/Inattention scale	6.29 (2.15)	
ADHD-RS Inattention scale	18.73 (5.67)	
ADHD-RS Hyperactivity/Impulsivity scale	12.90 (7.11)	
ADHD-RS Total Scale	31.62 (10.42)	
Medicated		148 (76.3)

Note. SES, highest educational level of the parents.

SDQ, Strengths and Difficulties Questionnaire.

ADHD-RS, ADHD rating scale.

### Confirmatory factor analyses

Confirmatory factor analyses using items from the three subscales of the READ were conducted using our sample of ADHD patients (*n* = 194). The 23-item model showed an acceptable model fit, *χ*^2^(227) = 495.790; CFI = .949; TLI = .943; RMSEA = .078, CI [.069, .088]. Standardized factor loadings are presented in Table 2.

**Table 2:** **Standardized factor loadings for the 23-item READ scale (*n*** **= 194)**

Item number and content	Standardized factor loadings
Personal Dispositions	
7 goal orientation items	0.76
12 realism items	0.55
17 competence items	0.77
20 self-confidence items	0.80
26 positive outlook items	0.69
2 aims and objectives	0.69
8 planfulness items	0.65
13 organizational skill items	0.65
6 positive social orientation items	0.76
11 making contact items	0.65
22 humor items	0.87
25 comforting others items	0.80
Family Cohesion	
5 shared values items	0.86
15 familial agreement items	0.81
10 comfort items	0.90
21 common positive outlook items	0.70
24 support items	0.86
27 shared activities items	0.72
Social Resources	
3 encouragement items	0.73
9 cohesion items	0.71
14 support items	0.82
19 help items	0.80
28 appreciated by others items	0.87

### Measurement model

The model showed an acceptable fit: CFI = .94; TLI = .93; RMSEA = .056, CI [.049, .063], with a significant chi-squared value, *χ*^2^(524) = 846.541, *p* = 0.000. The chi-squared/df-ratio was 1.62, which is commonly regarded as acceptable [55].

### Path models

#### Protective factors as mediators of ADHD

**Figure 2:**
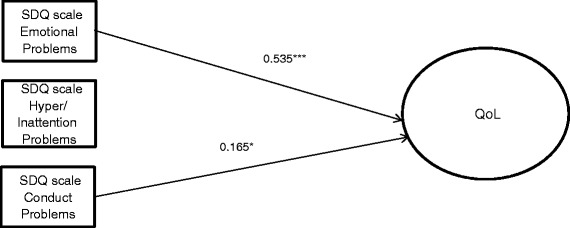
Path model with standardized estimates for direct effects without protective factors, adjusted for age and sex. Note. *p < .05, **p < .01, ***p < .001. SDQ – Strength and Difficulties Questionnaire.

A model analyzing the direct effect of coexisting problems on QoL was developed (see Figure 2). In this model, a higher level of Emotional Problems (β = 0.535) and Conduct Problems (β = 0.165) and Increasing Age (β = 0.143) decreased QoL.

**Figure 3:**
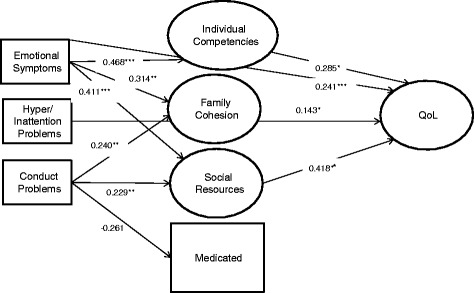
Final path model with standardized estimates adjusted for age and sex. Note. *p < .05, **p < .01, ***p < .001.

The final path model, which also included indirect effects of protective factors and medicated/unmedicated adolescents, is shown in Figure 3. Emotional Problems was mediated by Individual Competencies; thus, the direct effect on QoL (β = 0.535) was reduced in the final path model (β = 0.241). Similarly, Conduct Problems was mediated by Social Resources, thus diminishing the direct effect on QoL (β = 0.165). A higher level of Conduct Problems was associated with lower Family Cohesion (β = 0.240) and Social Resources (β = 0.229) and with being unmedicated (β = –0.261). A higher level of Emotional Problems was associated with lower Individual Competencies (β = 0.468), Family Cohesion (β = 0.314), and Social Resources (β = 0.411). A lower level of Individual Competencies (β = 0.285) and Social Resources (β = 0.418) was associated with a decreased QoL. Medical treatment was almost significantly associated with a better QoL in the present study (β = –0.150, *p* = 0.062). A higher level of Hyperactivity/Inattention was associated with decreased QoL when adjusted for all variables included in the final path model (β = 0.143). Increased Age was associated with decreased QoL (β = 0.120), similar to that observed in the direct effect model.

#### Protective factors as moderators: interaction model

Our analyses showed no interaction between coexisting problems (i.e., Emotional Problems, Conduct Problems, and Inattention/Hyperactivity) and protective factors (i.e., Individual Competencies, Family Cohesion, and Social Resources) and no effect on QoL. For example, Individual Competencies was not a moderator of the effect of Conduct Problems on QoL (β = –0.034, *p* = 0.644), of the effect of Emotional Problems on QoL (β = 0.005, *p* = 0.644), or of Hyperactivity/Inattention on QoL (β = –0.023, *p* = 0.221).

## Discussion

The aim of this study was to assess the role of protective factors as mediators and moderators of the relationship between coexisting emotional problems and conduct problems and QoL among adolescents with ADHD. The results, based on self-reports, showed that protective factors mediated the association between emotional and conduct problems and QoL, even after adjusting for prescribed medication. Individual Competencies was the strongest mediator of the relationship between coexisting emotional problems and QoL. Furthermore, we found no significant interactions between coexisting factors and individual competencies, which indicates that there were no moderators.

The major finding of this study was that individual competencies, which include structured style, social competence, and personal competence, were the strongest mediators of the relationship between emotional problems and QoL among adolescents with ADHD. These results suggest that adolescents with ADHD and a better structured style, social competence, and personal competence are more protected from coexisting emotional problems, and that these factors are associated with a better QoL. Executive function impairments among children with ADHD are heterogeneous [16,56]. The present study assessed organizational and planning skills, which might be particularly important during adolescence, whereas different aspects of executive functioning may be more important among younger children [57]. Another study indicated that planning and organizational abilities predicted academic functioning above and beyond the impact of ADHD symptoms [58]. It has been suggested that social competence mediates the relationship between ADHD and depression in children [59]. Moreover, personal competence may be an influential factor in everyday life, as it was found to mediate the relationship between ADHD symptoms and test anxiety [60] and to mediate partially the relationship between ADHD symptoms and adjustment to college [61]. Our results indicate that social competence and personal competence might also mediate the relationship between coexisting emotional problems and QoL. The use of individual competencies might allow the implementation of more targeted interventions aimed at improving coexisting problems and QoL. Structured style, social competence, and personal competence are considered plastic brain functions, and some studies have found that cognitive training is beneficial for individuals with ADHD [62-65], including studies of adults [66]. Furthermore, findings from the ADHD literature suggest that medical treatment significantly improves social functioning and self-esteem [19,67].

Our second finding was that social resources mediated the relationship between both emotional and conduct problems and QoL. Peer difficulties represent a significant area of impairment for adolescents with ADHD [13,68]. Our results indicate that ADHD patients with better social resources may be protected from coexisting emotional and conduct problems, and better Social Resources was associated with a greater QoL. Heiman [69] found that children with ADHD define friendship differently than do typically developing children. They tend to value certain characteristics in friendships that may conflict with those valued by their peer group, such as having fun compared with receiving emotional support; this can lead to a decreased likelihood of developing mutually satisfying friendships [70]. Longitudinal studies suggest that peer rejection predicts later negative outcomes, including emotional and conduct problems [2,20]. Moreover, according to McQuade et al. [71], being socially successful combined with modest perceptions of competence is a protective factor against behavioral problems. Several studies have found that coexisting conduct problems in children with ADHD severely worsen the adult outcome [6,7]. Therefore, supporting protective factors that attenuate the risk beyond the effect of medical treatment may be of great importance in the comprehensive treatment of these children and adolescents. Further research on these relationships is recommended

Another finding was the association between coexisting emotional and conduct problems and family cohesion. In a previous study, we found that coexisting problems had an impact on family functioning [23]. Better family functioning, as experienced by the adolescents with ADHD, was associated with fewer emotional and conduct problems. These findings are in line with recent research suggesting that higher family cohesion mediates the effect of foster care on children’s ADHD symptomatology [72]. Furthermore, positive development of executive functions, social competence, and peer outcomes has been associated with higher family cohesion, family functioning, and/or parent–child attachment during childhood [72,73]. We found no association between family cohesion and QoL; however, previous studies found that parental support was associated with QoL among college students with ADHD [74,75]. These differences in findings might be attributable to variations in the instruments used to measure QoL; in addition, the subjects included in the Grenwald-Mayes [74] and Wilmshurst et al. [75] studies were older than those reported in our study (mean age, 25 and 19 vs 15 years, respectively). The results of our study underline the importance of considering both individual and environmental factors in ADHD.

Finally, adolescents with ADHD who received medical treatment had fewer conduct problems, indicating a positive effect of medication on conduct problems, which is consistent with previous work [76]. Conversely, the level of emotional problems was unrelated to medication. The effect of medication on QoL did not quite reach statistical significance, which might have been caused by statistical power limitations. Some studies indicate that comorbid anxiety disorders are associated with a lower effect of medication on ADHD symptoms and psychosocial functioning, which leads to discontinuation of medical treatment [30]. A study of children and adolescents documented that, among treated individuals, about 6% were also treated for emotional disorders [77]. Some clinical samples of adolescents with ADHD have reported even higher levels of emotional problems [78]. In our sample, emotional problems included primarily coexisting problems, part of the ADHD symptomatology, or a side effect of medical treatment. However, the latter is somewhat less likely, because care is taken to minimize side effects [79]. Furthermore, our sample included a relatively high percentage of girls, who exhibit a higher prevalence of emotional problems during adolescence in both clinical and epidemiological studies [80,81].

The findings of the present study were limited by a low response rate, which could have led to imprecise results. Nevertheless, the reason for referral did not differ from the population of patients treated in the clinic during the study period. Another limitation was that the results were based only on self-reports. Previous studies have found that children with ADHD have a positive illusory bias and perceive their level of competence inaccurately [82,83]. Goodman [45] has described the sensitivity of the SDQ scale. The odds ratios for the emotional scale were similar for self-reports and parent reports, whereas the odds ratios for the conduct scale were higher in parent reports. This might indicate that conduct problems were underreported in the present study, which is in agreement with prior research [84]. Parent reports might have yielded different results regarding conduct problems. Conversely, self-report scales may increase awareness of internalizing problems [85]. A clinical interview with parents was not conducted, and the family structure was not assessed. Therefore, we were not able to adjust for parental ADHD or other chronic conditions in the analysis. Another limitation of the study was its cross-sectional design, which did not allow causal inferences based on the data. A longitudinal study would allow the assessment of reciprocal relationships between the variables, as well as the examination of the development of family functioning and QoL. Finally, the ADHD diagnosis was based on clinical *ICD-10* diagnoses rather than on standardized semistructured child psychiatric interviews. Interrater reliability scores were not available; however, all diagnoses were established by an experienced child and adolescent psychiatrist or a clinical psychologist, and were based on standard national and international guidelines. Furthermore, the mean scores of the ADHD-RS were in the same range as those reported by other studies, including those of Norwegian clinical samples of adolescents with ADHD [55,86].

## Conclusions

The current study provided new information regarding the role of protective factors in the relationship between ADHD and coexisting emotional and conduct problems, and regarding the impact of these factors on QoL. Individual competencies, family cohesion, and social resources may reduce emotional problems and behavioral problems and improve QoL among adolescents with ADHD and among medicated individuals. The assessment of protective factors, in addition to risk factors, may identify potential treatment goals.
